# The Conventional and Breakthrough Tool for the Study of L-Glutamate Transporters

**DOI:** 10.3390/membranes14040077

**Published:** 2024-03-27

**Authors:** Kanako Takahashi, Kaoru Sato

**Affiliations:** Laboratory of Neuropharmacology, Division of Pharmacology, National Institute of Health Sciences, Kanagawa 210-9501, Japan; ktakahashi@nihs.go.jp

**Keywords:** Xenopus oocyte, glutamate transporter, EAAT2, two-electrode voltage clamp (TEVC), overexpression, excitotoxicity

## Abstract

In our recent report, we clarified the direct interaction between the excitatory amino acid transporter (EAAT) 1/2 and polyunsaturated fatty acids (PUFAs) by applying electrophysiological and molecular biological techniques to Xenopus oocytes. Xenopus oocytes have a long history of use in the scientific field, but they are still attractive experimental systems for neuropharmacological studies. We will therefore summarize the pharmacological significance, advantages (especially in the study of EAAT2), and experimental techniques that can be applied to Xenopus oocytes; our new findings concerning L-glutamate (L-Glu) transporters and PUFAs; and the significant outcomes of our data. The data obtained from electrophysiological and molecular biological studies of Xenopus oocytes have provided us with further important questions, such as whether or not some PUFAs can modulate EAATs as allosteric modulators and to what extent docosahexaenoic acid (DHA) affects neurotransmission and thereby affects brain functions. Xenopus oocytes have great advantages in the studies about the interactions between molecules and functional proteins, especially in the case when the expression levels of the proteins are small in cell culture systems without transfections. These are also proper to study the mechanisms underlying the interactions. Based on the data collected in Xenopus oocyte experiments, we can proceed to the next step, i.e., the physiological roles of the compounds and their significances. In the case of EAAT2, the effects on the neurotransmission should be examined by electrophysiological approach using acute brain slices. For new drug development, pharmacokinetics pharmacodynamics (PKPD) data and blood brain barrier (BBB) penetration data are also necessary. In order not to miss the promising candidate compounds at the primary stages of drug development, we should reconsider using Xenopus oocytes in the early phase of drug development.

## 1. Basic Background on EAAT2 and Other L-Glutamate Transporters in the Central Nervous System

**Figure 1:**
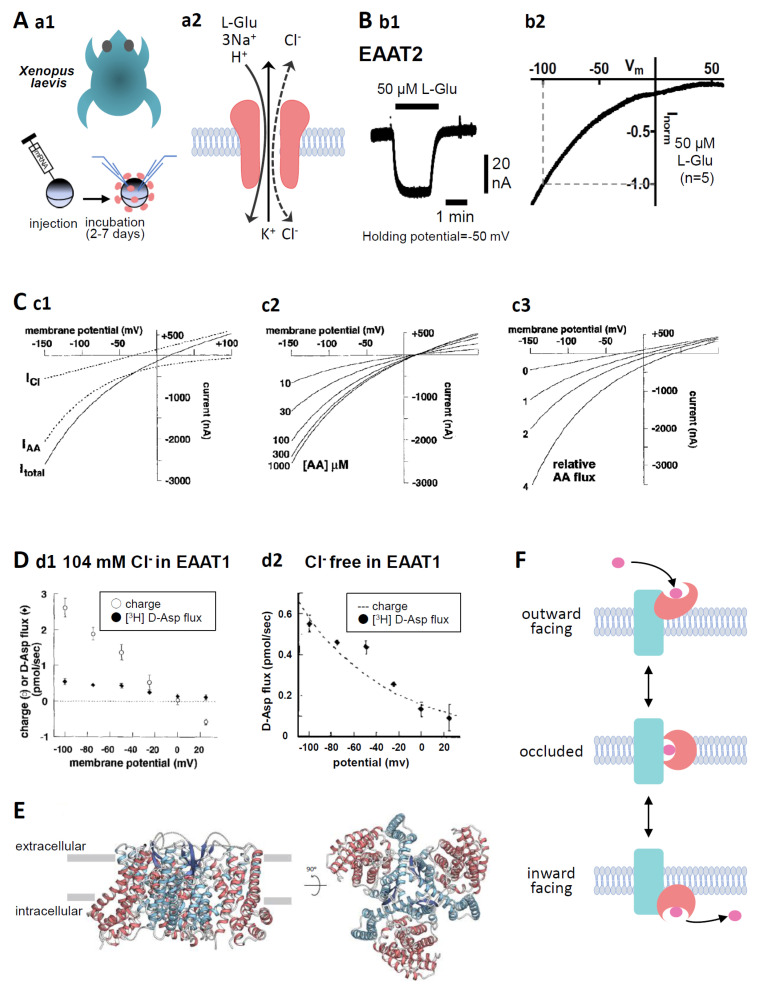
(**A**) (**a1**). Oocytes were collected from anaesthetized *Xenopus laevis*. The isolated oocytes were then treated with collagenase (2 mg mL^−1^, type 1), and capped mRNA was injected into either defolliculated stage V or VI oocytes. The oocytes were incubated for 2–7 d at 18 °C in ND96 solution containing 96 mM NaCl, 2 mM KCl, 1.8 mM CaCl_2_, 1 mM MgCl_2_, and 5 mM HEPES (pH 7.5) supplemented with 0.01% gentamycin. TEVC recordings from the oocytes were performed at room temperature (25 °C) using glass microelectrodes filled with 3 M KCl (resistance = 1–4 MΩ) and an Ag/AgCl pellet electrode. (**a2**). Substrate- and the coupling ion-transports by EAATs. Substrate, such as L-Glu, L-Asp, or D-Asp, transport through EAATs is coupled to the co-transport of 3 Na^+^ and 1 H^+^ followed by the counter transport of 1 K^+^. In addition, the binding of substrates and Na^+^ to EAATs activates uncoupled Cl^−^ anion currents. (**B**) (**b1**). A representative trace of L-Glu (50 μM for 2 min, black bar)-induced current obtained from Xenopus oocytes overexpressing EAAT2 clamped at −50 mV. (**b2**). IV relationship for L-Glu (50 μM)-induced EAAT2 current. To examine the IV relationship, the L-Glu-induced current was calculated through the subtraction of the steady-state current from the L-Glu-induced current. The curves were obtained with a holding potential of −60 mV applying an 8000 ms ramp pulse from −110 to +60 mV. Data are shown as the values normalized to that obtained with 50 μM L-Glu at −100 mV. Means, *n* = 5. (**C**) (**c1**). Model curves of transport-induced currents. The total L-Glu-induced EAAT currents (solid line: I_total_), electrophysiologically recorded using TEVC methods from EAAT-expressing Xenopus oocytes, represent the sum of the coupled L-Glu transport currents (dotted line: I_AA_) and the uncoupled Cl^−^ anion currents (dotted line: I_Cl_). (**c2**). The predicted reversal potential of the net current (I_total_) is independent of substrate concentration when the concentration dependence of I_AA_ and I_Cl_ is the same. However, the amplitude of I_total_ is dependent on the substrate concentration. (**c3**). The absolute reversal potential of I_total_ is dependent on I_AA_ relative to I_Cl_ [14] (copyright permission has been obtained). (**D**) (**d1**). D-Asp uptake and charge translocation were simultaneously measured during a 100 s application of 100 μM [^3^H] D-Asp to voltage-clamped oocytes expressing EAAT1 under 104 mM Cl^−^ conditions. (**d2**). Voltage dependence of RI-labelled D-Asp flux and superimposed exponential (e-fold/75 mV) derived from fit of transport current under nominal Cl^−^-free conditions [14] (copyright permission has been obtained). (**E**) Overall structure of human EAAT2 as viewed from the membrane plane (left) and the intracellular side (right). The trimerization domain is blue and the transport domain is red. The protomer of EAAT2 is divided into two distinct functional components: one is a rigid scaffold domain that mediates interprotomer interactions and is located in the center of the trimer, and the other is a transport domain containing the substrate-binding site [15]. (**F**) Schematic representation of the transport cycle of EAATs (elevator motion). The transport domain (red) moves across the membrane relative to the trimerization domain (blue). The transported L-Glu is pink. When L-Glu binds to the transport domain of the EAAT in outward facing state (top), the conformation changes to occluded state (middle) first, then changes to inward facing state (bottom), thereby releasing L-Glu into the cell.

Human excitatory amino acid transporters (EAATs) have five subtypes, EAAT1, 2, 3, 4, and 5 (EAAT1-3 [1], EAAT3 [2], EAAT4 [3], EAAT5 [4]). EAAT2 is the predominant excitatory neurotransmitter transporter that accounts for approximately 90% of L-glutamate (L-Glu) uptake in the forebrain [5], meaning that EAAT2 is essential for healthy brain functioning. In addition to refining synaptic transmission, one of the important roles of EAAT2 is protecting neurons from excitotoxicity, the neuron-specific cell death mechanism caused by prolonged elevation of extracellular L-Glu [6]. EAAT2 is expressed mainly in astrocytes, but 5 to 10% of the total EAAT2 expression is found in neurons [7]. The extracellular and intracellular electrochemical gradient of Na^+^ is a driving force of the transport. The principle of EAAT2 transport is as follows: 1 glutamate transport is coupled to the influx of 3 Na^+^ and 1 H^+^ followed by the outflux of 1 K^+^, resulting in electrogenicity [8] (Figure 1A(a2)). This coupling maintains a transmembrane L-Glu concentration gradient ([Glu]_in_/[Glu]_out_) exceeding 10^6^-fold under physiological conditions [8], i.e., the [Glu]_in_ of astrocytes is approximately 2–3 mM L-Glu in their cytoplasm, and [Glu]_out_ under resting-state conditions is approximately 25 nM [9]. Transport against the concentration gradient uses ATP derived from the Na^+^/K^+^-ATPase pathway [10].

EAAT2 is also related to neuronal energy metabolism [11,12]. When the intracellular Na^+^ concentration is changed in astrocytes, lactate is released from astrocytes to the neuron as the energy source. Furthermore, EAAT2-induced intracellular Na^+^ elevation drives the release of γ-aminobutyric acid (GABA) through the reversal of GABA transporter GAT-3, thereby resulting in tonic inhibition of neurons [13]. This mechanism provides an adjustable, in situ negative feedback on the excitability of neurons in physiological conditions.

## 2. EAAT2 Pharmacology

As described above, because EAAT2 is the predominant L-Glu transporter in the frontal cortex, defective EAAT2 transport leads to excitotoxic neuronal death [16,17,18,19], which has been identified as one of the pathological changes in Huntington’s disease [19,20], amyotrophic lateral sclerosis (ALS) [17,21], and schizophrenia [18]. EAAT2 has, therefore, become a therapeutic target in central nervous system drug development [22,23].

Despite the large demands for EAAT2-selective activators, they have not advanced beyond the clinical trial phase [23,24,25,26]. Table 1 shows widely used EAAT2 inhibitors and enhancers in this study field. These inhibitors were developed based on the structures of the substrates (L-Glu and L-Asp) and L-Glu analogues (α-amino-3-hydroxy-5-methyl-4-isoxazolepropionic acid [AMPA], kainic acid, etc.) as shown in the “extra info” column. Because orthosteric sites are well conserved among EAATs [27], many of the inhibitors interact with multiple EAATs. In these inhibitors, the substrate-type inhibitors (DL-threo-β-hydroxyaspartate [THA] [1,3,4] and L-trans-2.4-pyrrolidine dicarboxylate [L-trans-2.4-PDC] [1,3,4]) are transported in the same manner as the genuine substrates, causing the reduction of substrate uptake without any effects on ion flux, i.e., substrate-induced EAAT currents. Therefore, these inhibitors should not be used in the electrophysiological study. L-trans-2.3-PDC [28,29,30], (2S,4R)-4-methylglutamate (SYM2081) [31,32], DL-threo-β-benzyloxyaspartate (TBOA) [33,34,35], TFB-TBOA [36], dihydrokainic acid (DHK) [1,33], and N(4)-[4-(2-bromo-4,5-difluorophenoxy)phenyl]-L-asparagine (WAY213613) [37] are non-substrate-type inhibitors. TBOA and TFB-TBOA are broad blockers, while DHK and WAY213613 are EAAT2-selective blockers. Parawixin1 [38,39] and 3-((4-cyclohexylpiperazin-1-yl)(1-phenethyl-1H-tetrazol-5-yl)methyl)-6-methoxyquinolin-2(1H)-one (GTG949) [40] are allosteric-type EAAT2-selective enhancers. Parawixin1 was found in the venom extract of the spider *Parawixia bistriata*, and its structure has not been clarified. GTG949 was developed by virtual screening by adding functional groups to parawixin1. In addition to these direct modulators, ceftriaxone, a β-lactam antibiotic, enhances transport activity by increasing EAAT2 expression levels. Ceftriaxone increases EAAT2-mediated L-Glu uptake in rodent brains through an increase in EAAT2 expression levels [41] and is expected to be a breakthrough in the treatment of ALS. Despite promising preclinical data, ceftriaxone could not reveal significant effects in phase III clinical trials [42].

## 3. Reasons to Choose Xenopus Oocytes for the Study of EAAT2

Cells used for the pharmacological and physiological study of EAAT2 should meet the following two requirements: the cells should express enough EAAT2, and the molecular functions of the cells can be quantitatively investigated. Xenopus oocytes meet these two requirements and are appropriate for the study of EAAT2. We will explain this in the following paragraphs.

### 3.1. Advantages of Xenopus Oocytes That Overexpress EAAT2

EAAT1 and EAAT2 are mainly expressed in astrocytes [43,44], while EAAT2 is also expressed in neurons, but to a lesser extent. However, in the primary culture of astrocytes, the expression level of EAAT2 is very low [16,45], and the functional predominance switches from EAAT2 to EAAT1 [46,47,48,49]. In vitro substrate uptake assays are therefore performed using cell lines such as the Human Embryonic Kidney 293 (HEK 293) [50,51] and African green monkey kidney fibroblast-like (COS) cell lines [1,33], in which heterologous expression of cloned transporters has been induced. The difficulties of these in vitro assays are unignorable variations in the transfection ratios and the expression levels. In contrast, in terms of transfection, we inject the most probable amount of cRNA directly into Xenopus oocytes. Even though the expression levels depend on the target proteins, statistical analyses among experiments are possible [52]. The following are the additional technical advantages of the use of Xenopus oocytes:

▪ Direct injection enables to express the targeted proteins with a high success rate.
▪ Approximately 200 or more oocytes at defolliculated stage V or VI that are suitable for cRNA injection can be collected from one Xenopus. Four to six operations with 1-week intervals are possible per one Xenopus. Additionally, three injections are possible for the oocytes collected in one operation. Therefore, ready-to-use oocytes can be obtained 12 times a month.
▪ The transfection protocol is easy and feasible (Figure 1A(a1)). To overexpress the targeted protein in Xenopus oocytes, 50 nL of capped cRNA (10 ng) solution is directly injected into an oocyte by a nanoinjector installed with a glass microelectrode. An oocyte is a spherical cell with a diameter of 1–1.2 mm, large enough to confirm a successful injection by checking the swelling of the oocyte. Furthermore, the diameter of the microelectrode tip is 20–25 μm, which enables the minimization of membrane damage and the initiation of electrophysiological recording the next day.
▪ By modulating the interval between the injection and the analysis, the expression level of the targeted proteins can be regulated. Conversely, experiments using oocytes expressing almost the same level of the targeted proteins can be performed.
▪ The interactions of the targeted protein with the accessory subunits and/or modulatory proteins can be examined through the co-transfection of these proteins at the optimal ratio [e.g., protein interacting with C kinase 1 (PICK1), containing the postsynaptic density protein (PDZ) domain, with GLT1 (EAAT2 in rodents) [53]].

### 3.2. Measurement of the Activity of EAAT2 Expressed in Xenopus Oocytes

The quantification of the amount of radioisotope (RI)-labelled substrates transported into the oocytes is a conventionally and widely used protocol for the quantification of EAAT2 activity [24,54]. In the case of EAAT2, however, because the transport activity depends on the membrane potential, accurate quantification can be performed under the membrane potential clamp (Figure 1D) [14]. As described in Chapter 1, substrate transport is coupled to the influx of 3 Na^+^ and 1 H^+^ followed by the outflux of 1 K^+^, resulting in L-Glu transport-coupled currents (I_AA_) [8]. Furthermore, the uncoupled Cl^−^ anion channel is opened (I_Cl_) by substrate binding (Figure 1A(a2)). The model by Wadiche shows that the total L-Glu-induced EAAT currents (I_total_) are the sum of I_AA_ and I_Cl_ [14] (Figure 1C(c1)). Electrophysiological techniques allow real-time detection of the effects of compounds on L-Glu transport (Figure 1B(b1)). Furthermore, these techniques can also detect heteroexchange associated with transport, which is impossible to detect by RI-labelled substrate assay. The current–voltage (IV) relationships of I_AA_ show inward rectification with no reversal potential, that is the membrane potential at which the direction of ionic current reverses, while those of I_Cl_ show linear with reversal potential and outward current at positive membrane potentials. In I_total_, the outward current is due to I_Cl_ and the reversal potential is dependent on the relative magnitude of I_AA_ and I_Cl_ (Figure 1C(c3)). The amplitude of I_total_ is dependent on the amount of transported substrates, while the reversal potential of I_total_ is independent of the amount of substrates transported (Figure 1C(c2)).

EAAT1-5 differs in the proportion of I_AA_ and I_Cl_ in I_total_. The neuronal transporters EAAT4 and EAAT5 act primarily as Cl^−^ anion channels due to I_Cl_ dominance [3,4]. On the other hand, EAAT1, EAAT2, and EAAT3 have smaller I_Cl_ than I_AA_. Specifically, I_AA_ is predominant, and the contribution of the Cl^−^ anion current is very small in EAAT2 I_total_ [14]. In line with this, the open probability of Cl^−^ anion channels of EAAT2 transfected in HEK293 cells was confirmed to be 0.06 ± 0.01% [55]. We have confirmed that IV relationships for the L-Glu-induced EAAT2 current have no reversal potential up to +60 mV and are very similar to I_AA_ in Xenopus oocytes overexpressing EAAT2 by two-electrode whole-cell voltage clamp (TEVC) methods (Figure 1B(b2)). This result indicates that EAAT2 transport activity can be quantified by substrate-induced EAAT2 currents. In support of this, in the simultaneous recordings of the EAAT1 current and RI-labelled D-Asp uptake under Cl^−^ free conditions, EAAT1 current showed clear voltage dependence similar to D-Asp flux (Figure 1D) [14].

The following are the additional advantages of electrophysiological recording in Xenopus oocytes.

**Figure 2:**
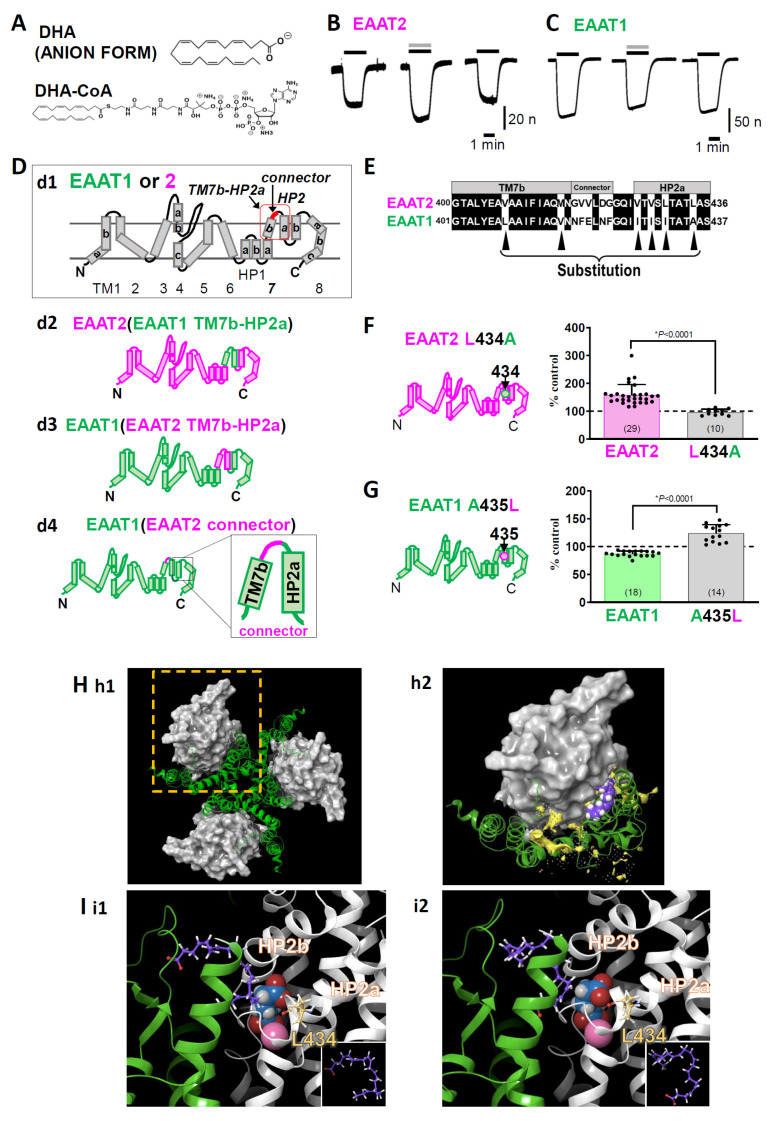
(**A**) The chemical structures of DHA and DHA-CoA. (**B**) Representative traces of L-glutamate (L-Glu, 50 μM for 2 min, black horizontal line)-induced current obtained from Xenopus oocytes overexpressing EAAT2 clamped at −50 mV in the absence or presence of DHA (100 μM for 2 min, grey horizontal line). When coapplied, DHA increased the L-Glu-induced EAAT2 current, and the effect disappeared after washout. (**C**) Representative traces of L-Glu-induced EAAT1 currents in the absence or presence of DHA. When the compounds were coapplied, DHA tended to decrease the EAAT1 current, and the effect disappeared after washout. (**D**) Topology of EAAT1, EAAT2 (**d1**). EAAT1/2 is organized into eight transmembrane (TM1-8) and two helical hairpins (HP1 and HP2), which are re-entrant loops. TM7b-HP2a sequence and connector sequence in the red square. d2-d4. EAAT1-EAAT2 hybrid chimeras: EAAT2 (EAAT1 TM7b-HP2a) (**d2**), EAAT1 (EAAT2 TM7b-HP2a) (**d3**), and EAAT1 (EAAT2 connector) (**d4**). (**E**) Amino acid alignment from TM7b to HP2a of EAAT2 and EAAT1. The common amino acids are shown on a black background. Single amino acid back-mutations were performed at the sites indicated by black arrowheads in the EAAT1 (EAAT2 TM7b-HP2a) chimaera. (**F**) Left, topology of EAAT2 L434A. Right, comparison of the effects of DHA on EAAT2 and EAAT2 L434A. Data are shown as rates of increase by DHA. (**G**) Left, topology of EAAT1 A435L. Right, comparison of the effects of DHA on EAAT1 and EAAT1 A435L. Data are shown as rates of increase by DHA. (**H**) Proposed binding conformation for DHA in the transport domain/scaffold domain interface of the EAAT2 homology model in the outward facing state (OFS). (**h1**). Extracellular view of the trimerized EAAT2 OFS homology model based on the EAAT1 crystal structure. The scaffold domain is shown as a green ribbon. The transport domain is shown as a grey surface. (**h2**). Magnified monomer in the hatched square in h1 in the presence of DHA. The lipid crevice calculated by SiteMap exists at the interface between the scaffold domain and the transport domain (yellow space). DHA is docked to the lipid crevice (carbon: purple spheres; hydrogen: white spheres). (**I**) Docking poses of DHA in the lipid pocket in the vicinity of HP2 according to the induced fit docking protocol. The scaffold domain and transport domain are shown in green and grey ribbons, respectively. The carbons in DHA and EAAT2 L434 are represented by purple and yellow sticks, respectively. The atoms in L-Glu are shown as follows: carbon, blue sphere; hydrogen, white sphere; oxygen, red sphere; nitrogen, hidden. Na^+^ is shown as a pink sphere. Two types of DHA conformations could be visualized according to the position of the carboxylic group, i.e., one with a carboxyl group on the upper side (**i1**) and the other with a carboxyl group on the lower side (**i2**). Both of them have similar U-shaped conformations. The inset shows the DHA conformations in each case. The three-dimensional position of DHA is in close proximity to the L-Glu binding site and Na^+^ binding site. Error bars represent the mean ±SD. The numbers written within parentheses in the respective figures represent the number of independent experiments. Statistical differences between groups were determined by two-tailed paired Student’s *t* test. *p* values are indicated in each figure panel [58].

▪ Measuring EAAT2 currents using electrophysiological techniques enable to detect the acute effects immediately (Figure 1B(b1) and Figure 2B,C), while the radioactivity detection of RI-labelled substrates has a time lag between the experiment and detection.
▪ Because a Xenopus oocyte is a large single cell, TEVC methods can be applied. Among the various types of electrophysiological techniques, it is rather easy to become proficient in performing TEVC. Furthermore, the oocyte membrane is so strong and stable that obtaining recordings from 10–30 oocytes per day and for longer than 30 min per oocyte is possible [53,56,57].
▪ Because the current values are so large (31.4 ± 21.7 nA, *n* = 27, holding potential = −50 mV) in Xenopus oocytes transfected with EAAT2 [58], accurate quantification of any modulation is possible.
▪ In addition to the whole oocyte clamp, more detailed experiments, such as single channel recording, outside-out patch clamp, and inside-out patch clamp, can be performed [59,60].
▪ It is possible to modify intracellular conditions in addition to the extracellular condition during recording. For example, it is possible to change intracellular conditions by filling glass microelectrodes with compounds such as H_2_O_2_ or DTT [57]. The extracellular conditions can be modulated by changing the pH, Cl^−^ concentration, etc. Furthermore, the chronic effects of compounds can be examined [61] through incubation with the conditioned medium for ~2 days.

### 3.3. Application of Molecular Biological Techniques to Xenopus Oocytes

To investigate the structural and functional properties of the targeted functional proteins in detail, site-directed mutagenesis, i.e., cysteine substitution, chimaera, etc., can be applied. The substituted cysteine accessibility method (SCAM) is typically used to investigate the structural information of membrane proteins [62,63]. In SCAM, local steric and electrostatic environments are probed by sulfhydryl reactive reagents because of the reactiveness of the substituted cysteines. This method has been applied to clarify the modulator binding sites and the regions related to the different functional states of many kinds of membrane proteins. The membrane topologies of EAATs have been studied by this method as well [64,65]. In addition, by inserting some pairs of cysteines and assaying their ability to form disulfide bonds, the proximity and mobility relationships between specific positions within the transporters have been clarified [66,67,68]. In Greek mythology, a chimaera is a monster that is a fusion of a lion, goat, and dragon. In molecular biology, a chimaera refers to cells or animals in which two or more genotypes are fused. In terms of the study of EAATs, quite a few chimaera combinations have been made to identify the binding sites for substrates and ions [69,70]. This technique was used to define the functional domains related to transport blockers. Vandenberg’s group defined the combination of helical hairpin (HP) 1, HP2, transmembrane (TM) 7, and TM8 to determine the sensitivity to transport blockers such as kainic acid [69] and 3-methylglutamate [70] (see Figure 2D about the membrane topology of EAATs).

## 4. New Findings about the Interactions between PUFAs and EAAT2 Obtained with Xenopus Oocyte Experiments

In this section, we will present our recent work regarding the modulation of EAAT2 function by docosahexaenoic acid (DHA) [58], in which electrophysiological and molecular biological techniques were applied to Xenopus oocytes overexpressing EAAT2. Because EAAT1 (GLAST in rodents) is also expressed in astrocytes in addition to EAAT2 (especially in the cerebellum) [71], we examined the effects of DHA (C22:6) (Figure 2A) and other polyunsaturated fatty acids (PUFAs) on EAAT2 and compared those on EAAT1. DHA has long been known to enhance synaptic transmission through multiple mechanisms, and L-Glu transporters are candidate proteins involved in target molecules.

We found that DHA significantly increased L-Glu-induced EAAT2 currents (Figure 2B) but slightly decreased L-Glu-induced EAAT1 currents (Figure 2C) using TEVC in Xenopus oocytes transfected with EAAT1 or EAAT2. Although there are some reports indicating the effects of DHA on EAATs, whether DHA interacts with EAATs directly has yet to be elucidated. To solve this problem, we attempted to identify the key amino acid for the DHA-EAAT2 interaction by EAAT1/2 chimaeras, focusing on the differences in the effects of DHA on EAAT1 and EAAT2. EAAT2 functions as a homotrimer, with each protomer having eight TM sites (TM1-8) and two HPs (HP1 and HP2), which are re-entrant loops (Figure 2D). The protomer is divided into two distinct functional components: one is a rigid scaffold domain (TM1, 2, 4, and 5) that mediates interprotomer interactions and is located in the center of the trimer, and the other is a transport domain (TM3, 6, 7, 8, HP1, and HP2) containing the substrate-binding site [15,72,73] (Figure 1E and Figure 2H). When we examined the effect of DHA-coenzyme A (DHA-CoA), a membrane-impermeable DHA analogue (Figure 2A), DHA-CoA increased the EAAT2 current almost to the same extent as DHA, suggesting that DHA approaches EAAT2 from the outside of cells. Therefore, we focused on the TM7b-HP2a sequence, the extracellular region of the transport domain (red square in Figure 2D(d1)). We first constructed EAAT2-based EAAT1/2 chimaeras. However, as sometimes occurs in chimaera experiments, the EAAT2-based chimaera for which its TM7b-HP2a was substituted to that of EAAT1 was nonfunctional (EAAT2 [EAAT1 TM7B-HP2a]) (Figure 2D(d2)). We therefore changed the chimaera from the EAAT2-based chimaera to the EAAT1-based chimaera. When the EAAT1 TM7b-HP2a region was replaced by that of EAAT2 (EAAT1 [EAAT2 TM7b-HP2a]) (Figure 2D(d3)), the effect of DHA on the L-Glu current was enhanced. We first suspected that the least conserved region in TM7b-HP2a, the “connector” in Figure 2D(d4),E, could cause the differences in responses between EAAT1 and EAAT2. When we replaced the “connecter” sequence of EAAT1 with that of EAAT2, i.e., in EAAT1 (EAAT2 connecter), the augmentative effect of DHA was not induced. We therefore focused on the amino acids in TM7b-HP2 other than the “connector”. Six amino acids (Val407, Met415, Val426, Val428, Leu430, and Leu434 in EAAT2) in TM7b-HP2a are different between EAAT1 and EAAT2 (black arrowheads in Figure 2E), so we back-mutated the above six amino acids one by one to the original EAAT1 amino acid. Among the mutants, only L434A showed a complete disappearance of the effect of DHA. We confirmed that a single mutation of Leu434 in WT EAAT2 to Ala completely suppressed the effect of DHA (Figure 2F). Furthermore, a single mutation of Ala435 in WT EAAT1 (corresponding to EAAT2 Leu434) to Leu also changed the effect from inhibition to enhancement (Figure 2G). The data above strongly suggest the direct interaction of DHA with EAAT2.

To confirm the feasibility of our hypothesis, we next performed a docking simulation between DHA and EAAT2. The dynamics of the scaffold domain and transport domain are involved in the substrate transport processes of L-Glu transporters. The first transport concept was developed by Jardetzky as an alternating access model in 1966 [74]: outward facing state (OFS), occluded state, and inward facing state (IFS). The structural basis for the transporter function is the elevator movement (Figure 1F) of the scaffold domain/transport domain backed up by the four crystal structures of Glt_Ph_ [OFS: [72,75] → intermediate (i) OFS: [76] → unlocked IFS: [77] → IFS: [78]]. We performed a docking analysis of DHA and EAAT2 OFS using EAAT1 as a template (protein data bank ID [PDBID]: 5LLM) [79]. Among seven candidate combinations of docking sites and DHA poses, which were proposed by a standard-induced fit docking protocol [80,81,82], the two top-scoring combinations are shown in Figure 2H,I. By performing calculations with SiteMap (SiteMap, Schrödinger, LLC, New York, USA), we found that DHA was docked in the lipid crevice, facing the interface of the transport domain/scaffold domain, which is close to the binding sites of the substrate and Na^+^. The DHA poses are U-shaped in both cases (Figure 2I) [58].

## 5. New Insights Suggested by the Interactions between DHA and L-Glu Transporters

### 5.1. Some PUFAs Modulate EAATs as Allosteric Modulators

Docking analysis of EAAT2 and DHA suggests that the most stable docking site is located at the transport domain/scaffold domain interface close to the binding sites of the substrates and Na^+^. Some allosteric modulators, such as GTG949, an EAAT2 selective enhancer [40], and UCPH101, an EAAT1 selective blocker [79,83], interact with the transport domain/scaffold domain interface and affect the movement of the transport domain, namely, elevator motion [79]. Detailed structural studies on Glt_Ph_, an archaeal EAAT homologue of the thermophilic prokaryote *Pyrococcus horikoshii*, provide supportive evidence for the direct interactions between transporters and lipids, including PUFAs [75,77,84]. In addition, the crystal structure analysis of Glt_Ph_ in the unlocked IFS suggests that the binding of lipids to the interface may facilitate the sliding of the transport domain. Most recently, cryo-electron microscopy (cryoEM) analysis has provided evidence for the direct interactions of EAAT2 and lipids [73]. Our data and recent related findings suggest that DHA allosterically regulates EAAT2 by enhancing the sliding of the transport domain. Furthermore, we also clarified that among 10 fatty acids [docosapentaenoic acid (DPA, C22:5), docosatetraenoic acid (C22:4), docosatrienoic acid (C22:3), eicosapentaenoic acid (EPA, C20:5), arachidonic acid, (ARA, C20:4), eicosatrienoic acid (C20:3), eicosadienoic acid (C20:2), α-linolenic acid (ALA, C18:3), linoleic acid (C18:2), and oleic acid (C18:1)], only DPA, EPA, ARA, and ALA showed augmentative effects on EAAT2 and slight inhibition of EAAT1 through the same mechanisms that require interactions with Leu434. Our data further suggest that a particular three-dimensional structure of PUFAs leads to the allosteric modulation of EAAT2.

### 5.2. Physiological Significance: The Potential of DHA as a Neurotransmission Modulator

The interactions between PUFAs and synaptic transmission were previously reported for ARA (ω-6, C20:4) [85,86,87]. However, there remain many PUFAs whose effects on synaptic transmission are unknown. DHA is the most abundant ω-3 PUFA in the mammalian brain [88,89,90] and the major constituent of membrane phospholipids [91]. DHA is synthesized in astrocytes from precursor fatty acids [92,93,94] and supplied to neuronal membranes as well. Under stable conditions, DHA is esterified to phosphatidylserine or phosphatidylethanolamine. When L-Glu stimulates neurons and astrocytes via L-Glu receptors [95,96], Ca^2+^-independent phospholipase A2 (iPLA2) is activated, thereby triggering DHA release [97,98]. The previous data and our findings raise the possibility that endogenous DHA modulates excitatory synaptic transmission. Some studies have reported favorable roles for DHA in human cognition and behavior; however, consistent conclusions have not been obtained [99]. In animal models and in vitro models, supporting data have been shown. EAATs control synaptic transmission, neurotransmitter spillover, and long-term plasticity [100,101,102,103]. Furthermore, DHA facilitates corticostriatal long-term potentiation (LTP) [104], the basic mechanisms for learning and memory. In support of this, selective inhibition of iPLA2 abolishes the expression of LTP [105], and DHA recovered the abolishment of LTP.

## 6. Summary

We could clarify the direct interaction between EAAT1/2 and DHA by applying electrophysiological and molecular biological techniques to Xenopus oocytes. Furthermore, we could make the difference between EAAT1 and EAAT2 clear. Our results indicate that Xenopus oocytes have great advantages in the studies about the interactions between molecules and EAATs, especially in the case when the expression levels are small in cell culture systems without transfections. Xenopus oocytes are also proper to study the mechanisms underlying the interactions, i.e., the docking sites, allosteric modulation, etc. Based on these data, we can proceed the study to the next step. The physiological role and significance can be investigated. In the case of EAAT2, the effects on the neurotransmission should be examined by electrophysiological approach using acute brain slices, etc. For new drug development, PKPD data and BBB penetration should also be needed. In order not to miss promising candidate compounds, we should reconsider using Xenopus oocytes in the early phase of drug development.

## Figures and Tables

**Table 1:** Pharmacological profiles of EAAT2 inhibitors and enhancers. The values in the selectivity column represent Kd for substrates, Ki for blockers, and EC50 for enhancers for the respective EAAT subtypes. The arrows in “effect on EAAT2 current” column represent whether the effect is inhibition (downward) or enhancement (upward). - means no effects.

	Compounds	Selectivity	Type for EAAT2	Effect on EAAT2 Current	Extra Info	Refs
**Inhibitors**	**THA** DL-threo-β-hydroxyaspartate	Substrate for EAAT1-4 EAAT1 = 32 μM EAAT2 = 19 μM EAAT3 = 25 μM Blocker for EAAT5 EAAT5 = 1 μM	**Substrate**	**-**	Aspartate derivative Weak NMDA receptor antagonist	[1,3,4]
	**L-trans-2.4-PDC** L-trans-2.4-pyrrolidine dicarboxylate	Substrate for EAAT1-4 EAAT1 = 79 μM EAAT2 = 8 μM EAAT3 = 61 μM EAAT4 = 2.6 μM Blocker for EAAT5 EAAT5 = 6 μM	**Substrate**	**-**	Pyrrolidine dicarboxylate derivative	[1,3,4]
	**L-trans-2.3-PDC** L-trans-2.3-pyrrolidine dicarboxylate	Blocker for EAAT2 EAAT2 = 12 μM	**Blocker**	**↓**	Pyrrolidine dicarboxylate derivative	[28,29,30]
	**SYM2081** (2S,4R)-4-methylglutamate	Substrate for EAAT1 EAAT1 = 54 μM Blocker for EAAT2 EAAT2 = 3.4 μM	**Blocker**	**↓**	Methyl-substituted glutamate Agonist for kainite receptor (IC_50_ = 35 nM)	[31,32]
	**TBOA** DL-threo-β-benzyloxyaspartate	Broad blocker [37] EAAT1 = 70 μM EAAT2 = 6 μM EAAT3 = 6 μM EAAT4 = 4.4 μM EAAT5 = 3.2 μM	**Blocker**	**↓**	Aspartate derivative	[33,34,35]
	**TFB-TBOA** (2S,3S)-3-{3-[4-(trifluoromethyl)benzoylamino]benzyloxy}aspartate	Broad blocker EAAT1 = 22 nM EAAT2 = 17 nM EAAT3 = 300 nM	**Blocker**	**↓**	Aspartate derivative Relatively weak affinity toward NMDA receptor (IC_50_ = 49 μM)	[36]
	**DHK** Dihydrokainic acid	Selective EAAT2 blocker EAAT1 > 3 mM EAAT2 = 23 μM EAAT3 > 3 mM	**Blocker**	**↓**	Kainate derivative Agonist for kainite receptor (IC_50_ = 6 μM)	[1,33]
	**WAY213613** N(4)-[4-(2-bromo-4,5-difluorophenoxy)phenyl]-L-asparagine	Potent EAAT2 blocker EAAT1 = 5 μM EAAT2 = 85 nM EAAT3 = 3.8 μM	**Blocker**	**↓**	Aspartate derivative	[37]
**Enhancers**	**Parawixin1**	Selective EAAT2 enhancer	**Allosteric**	**↑**	The venom extract of the spider *Parawixia bistriata*	[38,39]
	**GT949** 3-((4-cyclohexylpiperazin-1-yl)(1-phenethyl-1H-tetrazol-5-yl)methyl)-6-methoxyquinolin-2(1H)-one	Selective EAAT2 enhancer EAAT2= 0.26 nM	**Allosteric**	**↑**		[40]

## Data Availability

The original contributions presented in the study are included in the article, further inquiries can be directed to the corresponding author.

## Abbreviations

ALS	Amyotrophic lateral sclerosis
DHA	Docosahexaenoic acid
EAAT	Excitatory amino acid transporter
HP	Helical hairpin
IFS	Inward facing state
iPLA2	Ca^2+^-independent phospholipase A2
IV relation	Current–voltage relation
L-Asp	Aspartate
L-Glu	Glutamate
OFS	Outward facing state
PUFA	Polyunsaturated fatty acid
SCAM	Substituted cysteine accessibility method
TEVC	Two-electrode whole cell voltage clamp
TM	Transmembrane

## References

[1] Arriza J.L., Fairman W.A., Wadiche J.I., Murdoch G.H., Kavanaugh M.P., Amara S.G. (1994). Functional comparisons of three glutamate transporter subtypes cloned from human motor cortex. J. Neurosci..

[2] Kanai Y., Hediger M.A. (1992). Primary structure and functional characterization of a high-affinity glutamate transporter. Nature.

[3] Fairman W.A., Vandenberg R.J., Arriza J.L., Kavanaugh M.P., Amara S.G. (1995). An excitatory amino-acid transporter with properties of a ligand-gated chloride channel. Nature.

[4] Arriza J.L., Eliasof S., Kavanaugh M.P., Amara S.G. (1997). Excitatory amino acid transporter 5, a retinal glutamate transporter coupled to a chloride conductance. Proc. Natl. Acad. Sci. USA.

[5] Tanaka K., Watase K., Manabe T., Yamada K., Watanabe M., Takahashi K., Iwama H., Nishikawa T., Ichihara N., Kikuchi T. (1997). Epilepsy and exacerbation of brain injury in mice lacking the glutamate transporter GLT-1. Science.

[6] Michaelis E.K. (1998). Molecular biology of glutamate receptors in the central nervous system and their role in excitotoxicity, oxidative stress and aging. Prog. Neurobiol..

[7] Mennerick S., Dhond R.P., Benz A., Xu W., Rothstein J.D., Danbolt N.C., Isenberg K.E., Zorumski C.F. (1998). Neuronal expression of the glutamate transporter GLT-1 in hippocampal microcultures. J. Neurosci..

[8] Zerangue N., Kavanaugh M.P. (1996). Flux coupling in a neuronal glutamate transporter. Nature.

[9] Herman M.A., Jahr C.E. (2007). Extracellular glutamate concentration in hippocampal slice. J. Neurosci..

[10] Rose E.M., Koo J.C., Antflick J.E., Ahmed S.M., Angers S., Hampson D.R. (2009). Glutamate transporter coupling to Na,K-ATPase. J. Neurosci..

[11] Voutsinos-Porche B., Bonvento G., Tanaka K., Steiner P., Welker E., Chatton J.Y., Magistretti P.J., Pellerin L. (2003). Glial glutamate transporters mediate a functional metabolic crosstalk between neurons and astrocytes in the mouse developing cortex. Neuron.

[12] Magistretti P.J., Allaman I. (2015). A cellular perspective on brain energy metabolism and functional imaging. Neuron.

[13] Héja L., Nyitrai G., Kékesi O., Dobolyi A., Szabó P., Fiáth R., Ulbert I., Pál-Szenthe B., Palkovits M., Kardos J. (2012). Astrocytes convert network excitation to tonic inhibition of neurons. BMC Biol..

[14] Wadiche J.I., Amara S.G., Kavanaugh M.P. (1995). Ion fluxes associated with excitatory amino acid transport. Neuron.

[15] Kato T., Kusakizako T., Jin C., Zhou X., Ohgaki R., Quan L., Xu M., Okuda S., Kobayashi K., Yamashita K. (2022). Structural insights into inhibitory mechanism of human excitatory amino acid transporter EAAT2. Nat. Commun..

[16] Takaki J., Fujimori K., Miura M., Suzuki T., Sekino Y., Sato K. (2012). L-glutamate released from activated microglia downregulates astrocytic L-glutamate transporter expression in neuroinflammation: The ‘collusion’ hypothesis for increased extracellular L-glutamate concentration in neuroinflammation. J. Neuroinflamm..

[17] Rothstein J.D., Van Kammen M., Levey A.I., Martin L.J., Kuncl R.W. (1995). Selective loss of glial glutamate transporter GLT-1 in amyotrophic lateral sclerosis. Ann. Neurol..

[18] Heider J., Vogel S., Volkmer H., Breitmeyer R. (2021). Human iPSC-Derived Glia as a Tool for Neuropsychiatric Research and Drug Development. Int. J. Mol. Sci..

[19] Wilton D.K., Stevens B. (2020). The contribution of glial cells to Huntington’s disease pathogenesis. Neurobiol. Dis..

[20] Garcia V.J., Rushton D.J., Tom C.M., Allen N.D., Kemp P.J., Svendsen C.N., Mattis V.B. (2019). Huntington’s Disease Patient-Derived Astrocytes Display Electrophysiological Impairments and Reduced Neuronal Support. Front. Neurosci..

[21] Tyzack G., Lakatos A., Patani R. (2016). Human Stem Cell-Derived Astrocytes: Specification and Relevance for Neurological Disorders. Curr. Stem Cell Rep..

[22] Hinoi E., Takarada T., Tsuchihashi Y., Yoneda Y. (2005). Glutamate transporters as drug targets. Curr. Drug Targets CNS Neurol. Disord..

[23] Lin L., Yee S.W., Kim R.B., Giacomini K.M. (2015). SLC transporters as therapeutic targets: Emerging opportunities. Nat. Rev. Drug Discov..

[24] Wang W.W., Gallo L., Jadhav A., Hawkins R., Parker C.G. (2020). The Druggability of Solute Carriers. J. Med. Chem..

[25] Bunch L., Erichsen M.N., Jensen A.A. (2009). Excitatory amino acid transporters as potential drug targets. Expert Opin. Ther. Targets.

[26] Bridges R.J., Esslinger C.S. (2005). The excitatory amino acid transporters: Pharmacological insights on substrate and inhibitor specificity of the EAAT subtypes. Pharmacol. Ther..

[27] Vandenberg R.J., Ryan R.M. (2013). Mechanisms of glutamate transport. Physiol. Rev..

[28] Koch H.P., Kavanaugh M.P., Esslinger C.S., Zerangue N., Humphrey J.M., Amara S.G., Chamberlin A.R., Bridges R.J. (1999). Differentiation of substrate and nonsubstrate inhibitors of the high-affinity, sodium-dependent glutamate transporters. Mol. Pharmacol..

[29] Willis C.L., Humphrey J.M., Koch H.P., Hart J.A., Blakely T., Ralston L., Baker C.A., Shim S., Kadri M., Chamberlin A.R. (1996). L-trans-2,3-pyrrolidine dicarboxylate: Characterization of a novel excitotoxin. Neuropharmacology.

[30] Bridges R.J., Kavanaugh M.P., Chamberlin A.R. (1999). A pharmacological review of competitive inhibitors and substrates of high-affinity, sodium-dependent glutamate transport in the central nervous system. Curr. Pharm. Des..

[31] Vandenberg R.J., Mitrovic A.D., Chebib M., Balcar V.J., Johnston G.A. (1997). Contrasting modes of action of methylglutamate derivatives on the excitatory amino acid transporters, EAAT1 and EAAT2. Mol. Pharmacol..

[32] Donevan S.D., Beg A., Gunther J.M., Twyman R.E. (1998). The methylglutamate, SYM 2081, is a potent and highly selective agonist at kainate receptors. J. Pharmacol. Exp. Ther..

[33] Shimamoto K., Lebrun B., Yasuda-Kamatani Y., Sakaitani M., Shigeri Y., Yumoto N., Nakajima T. (1998). DL-threo-beta-benzyloxyaspartate, a potent blocker of excitatory amino acid transporters. Mol. Pharmacol..

[34] Shigeri Y., Shimamoto K., Yasuda-Kamatani Y., Seal R.P., Yumoto N., Nakajima T., Amara S.G. (2001). Effects of threo-beta-hydroxyaspartate derivatives on excitatory amino acid transporters (EAAT4 and EAAT5). J. Neurochem..

[35] Jabaudon D., Shimamoto K., Yasuda-Kamatani Y., Scanziani M., Gähwiler B.H., Gerber U. (1999). Inhibition of uptake unmasks rapid extracellular turnover of glutamate of nonvesicular origin. Proc. Natl. Acad. Sci. USA.

[36] Shimamoto K., Sakai R., Takaoka K., Yumoto N., Nakajima T., Amara S.G., Shigeri Y. (2004). Characterization of novel L-threo-beta-benzyloxyaspartate derivatives, potent blockers of the glutamate transporters. Mol. Pharmacol..

[37] Dunlop J., McIlvain H.B., Carrick T.A., Jow B., Lu Q., Kowal D., Lin S., Greenfield A., Grosanu C., Fan K. (2005). Characterization of novel aryl-ether, biaryl, and fluorene aspartic acid and diaminopropionic acid analogs as potent inhibitors of the high-affinity glutamate transporter EAAT2. Mol. Pharmacol..

[38] Fontana A.C., de Oliveira Beleboni R., Wojewodzic M.W., Ferreira Dos Santos W., Coutinho-Netto J., Grutle N.J., Watts S.D., Danbolt N.C., Amara S.G. (2007). Enhancing glutamate transport: Mechanism of action of Parawixin1, a neuroprotective compound from Parawixia bistriata spider venom. Mol. Pharmacol..

[39] Fontana A.C., Guizzo R., de Oliveira Beleboni R., Meirelles E.S.A.R., Coimbra N.C., Amara S.G., dos Santos W.F., Coutinho-Netto J. (2003). Purification of a neuroprotective component of Parawixia bistriata spider venom that enhances glutamate uptake. Br. J. Pharmacol..

[40] Kortagere S., Mortensen O.V., Xia J., Lester W., Fang Y., Srikanth Y., Salvino J.M., Fontana A.C.K. (2018). Identification of Novel Allosteric Modulators of Glutamate Transporter EAAT2. ACS Chem. Neurosci..

[41] Rothstein J.D., Patel S., Regan M.R., Haenggeli C., Huang Y.H., Bergles D.E., Jin L., Dykes Hoberg M., Vidensky S., Chung D.S. (2005). Beta-lactam antibiotics offer neuroprotection by increasing glutamate transporter expression. Nature.

[42] Cudkowicz M.E., Titus S., Kearney M., Yu H., Sherman A., Schoenfeld D., Hayden D., Shui A., Brooks B., Conwit R. (2014). Safety and efficacy of ceftriaxone for amyotrophic lateral sclerosis: A multi-stage, randomised, double-blind, placebo-controlled trial. Lancet Neurol..

[43] Lehre K.P., Levy L.M., Ottersen O.P., Storm-Mathisen J., Danbolt N.C. (1995). Differential expression of two glial glutamate transporters in the rat brain: Quantitative and immunocytochemical observations. J. Neurosci..

[44] Haugeto O., Ullensvang K., Levy L.M., Chaudhry F.A., Honoré T., Nielsen M., Lehre K.P., Danbolt N.C. (1996). Brain glutamate transporter proteins form homomultimers. J. Biol. Chem..

[45] Sato K., Matsuki N., Ohno Y., Nakazawa K. (2003). Estrogens inhibit l-glutamate uptake activity of astrocytes via membrane estrogen receptor alpha. J. Neurochem..

[46] Gegelashvili G., Danbolt N.C., Schousboe A. (1997). Neuronal soluble factors differentially regulate the expression of the GLT1 and GLAST glutamate transporters in cultured astroglia. J. Neurochem..

[47] Schlag B.D., Vondrasek J.R., Munir M., Kalandadze A., Zelenaia O.A., Rothstein J.D., Robinson M.B. (1998). Regulation of the glial Na+-dependent glutamate transporters by cyclic AMP analogs and neurons. Mol. Pharmacol..

[48] Swanson R.A., Liu J., Miller J.W., Rothstein J.D., Farrell K., Stein B.A., Longuemare M.C. (1997). Neuronal regulation of glutamate transporter subtype expression in astrocytes. J. Neurosci..

[49] Yang Y., Gozen O., Watkins A., Lorenzini I., Lepore A., Gao Y., Vidensky S., Brennan J., Poulsen D., Won Park J. (2009). Presynaptic regulation of astroglial excitatory neurotransmitter transporter GLT1. Neuron.

[50] Berry C.B., Hayes D., Murphy A., Wiessner M., Rauen T., McBean G.J. (2005). Differential modulation of the glutamate transporters GLT1, GLAST and EAAC1 by docosahexaenoic acid. Brain Res..

[51] Dunlop J., Lou Z., Zhang Y., McIlvain H.B. (1999). Inducible expression and pharmacology of the human excitatory amino acid transporter 2 subtype of L-glutamate transporter. Br. J. Pharmacol..

[52] Duffield M., Patel A., Mortensen O.V., Schnur D., Gonzalez-Suarez A.D., Torres-Salazar D., Fontana A.C.K. (2020). Transport rate of EAAT2 is regulated by amino acid located at the interface between the scaffolding and substrate transport domains. Neurochem. Int..

[53] Sogaard R., Borre L., Braunstein T.H., Madsen K.L., MacAulay N. (2013). Functional modulation of the glutamate transporter variant GLT1b by the PDZ domain protein PICK1. J. Biol. Chem..

[54] Dvorak V., Wiedmer T., Ingles-Prieto A., Altermatt P., Batoulis H., Bärenz F., Bender E., Digles D., Dürrenberger F., Heitman L.H. (2021). An Overview of Cell-Based Assay Platforms for the Solute Carrier Family of Transporters. Front. Pharmacol..

[55] Kolen B., Kortzak D., Franzen A., Fahlke C. (2020). An amino-terminal point mutation increases EAAT2 anion currents without affecting glutamate transport rates. J. Biol. Chem..

[56] Trotti D., Danbolt N.C., Volterra A. (1998). Glutamate transporters are oxidant-vulnerable: A molecular link between oxidative and excitotoxic neurodegeneration?. Trends Pharmacol. Sci..

[57] Trotti D., Rolfs A., Danbolt N.C., Brown R.H., Hediger M.A. (1999). SOD1 mutants linked to amyotrophic lateral sclerosis selectively inactivate a glial glutamate transporter. Nat. Neurosci..

[58] Takahashi K., Chen L., Sayama M., Wu M., Hayashi M.K., Irie T., Ohwada T., Sato K. (2022). Leucine 434 is essential for docosahexaenoic acid-induced augmentation of L-glutamate transporter current. J. Biol. Chem..

[59] Otis T.S., Kavanaugh M.P. (2000). Isolation of current components and partial reaction cycles in the glial glutamate transporter EAAT2. J. Neurosci..

[60] Wadiche J.I., Kavanaugh M.P. (1998). Macroscopic and microscopic properties of a cloned glutamate transporter/chloride channel. J. Neurosci..

[61] Fairman W.A., Sonders M.S., Murdoch G.H., Amara S.G. (1998). Arachidonic acid elicits a substrate-gated proton current associated with the glutamate transporter EAAT4. Nat. Neurosci..

[62] Akabas M.H. (2015). Cysteine Modification: Probing Channel Structure, Function and Conformational Change. Adv. Exp. Med. Biol..

[63] Akabas M.H., Stauffer D.A., Xu M., Karlin A. (1992). Acetylcholine receptor channel structure probed in cysteine-substitution mutants. Science.

[64] Grunewald M., Bendahan A., Kanner B.I. (1998). Biotinylation of single cysteine mutants of the glutamate transporter GLT-1 from rat brain reveals its unusual topology. Neuron.

[65] Grunewald M., Menaker D., Kanner B.I. (2002). Cysteine-scanning mutagenesis reveals a conformationally sensitive reentrant pore-loop in the glutamate transporter GLT-1. J. Biol. Chem..

[66] Rong X., Tan F., Wu X., Zhang X., Lu L., Zou X., Qu S. (2016). TM4 of the glutamate transporter GLT-1 experiences substrate-induced motion during the transport cycle. Sci. Rep..

[67] Zhang Y., Kanner B.I. (1999). Two serine residues of the glutamate transporter GLT-1 are crucial for coupling the fluxes of sodium and the neurotransmitter. Proc. Natl. Acad. Sci. USA.

[68] Brocke L., Bendahan A., Grunewald M., Kanner B.I. (2002). Proximity of two oppositely oriented reentrant loops in the glutamate transporter GLT-1 identified by paired cysteine mutagenesis. J. Biol. Chem..

[69] Vandenberg R.J., Arriza J.L., Amara S.G., Kavanaugh M.P. (1995). Constitutive ion fluxes and substrate binding domains of human glutamate transporters. J. Biol. Chem..

[70] Mitrovic A.D., Amara S.G., Johnston G.A., Vandenberg R.J. (1998). Identification of functional domains of the human glutamate transporters EAAT1 and EAAT2. J. Biol. Chem..

[71] Storck T., Schulte S., Hofmann K., Stoffel W. (1992). Structure, expression, and functional analysis of a Na(+)-dependent glutamate/aspartate transporter from rat brain. Proc. Natl. Acad. Sci. USA.

[72] Yernool D., Boudker O., Jin Y., Gouaux E. (2004). Structure of a glutamate transporter homologue from *Pyrococcus horikoshii*. Nature.

[73] Zhang Z., Chen H., Geng Z., Yu Z., Li H., Dong Y., Zhang H., Huang Z., Jiang J., Zhao Y. (2022). Structural basis of ligand binding modes of human EAAT2. Nat. Commun..

[74] Jardetzky O. (1966). Simple allosteric model for membrane pumps. Nature.

[75] Boudker O., Ryan R.M., Yernool D., Shimamoto K., Gouaux E. (2007). Coupling substrate and ion binding to extracellular gate of a sodium-dependent aspartate transporter. Nature.

[76] Verdon G., Boudker O. (2012). Crystal structure of an asymmetric trimer of a bacterial glutamate transporter homolog. Nat. Struct. Mol. Biol..

[77] Akyuz N., Georgieva E.R., Zhou Z., Stolzenberg S., Cuendet M.A., Khelashvili G., Altman R.B., Terry D.S., Freed J.H., Weinstein H. (2015). Transport domain unlocking sets the uptake rate of an aspartate transporter. Nature.

[78] Reyes N., Ginter C., Boudker O. (2009). Transport mechanism of a bacterial homologue of glutamate transporters. Nature.

[79] Canul-Tec J.C., Assal R., Cirri E., Legrand P., Brier S., Chamot-Rooke J., Reyes N. (2017). Structure and allosteric inhibition of excitatory amino acid transporter 1. Nature.

[80] Sherman W., Beard H.S., Farid R. (2006). Use of an induced fit receptor structure in virtual screening. Chem. Biol. Drug Des..

[81] Sherman W., Day T., Jacobson M.P., Friesner R.A., Farid R. (2006). Novel procedure for modeling ligand/receptor induced fit effects. J. Med. Chem..

[82] Friesner R.A., Banks J.L., Murphy R.B., Halgren T.A., Klicic J.J., Mainz D.T., Repasky M.P., Knoll E.H., Shelley M., Perry J.K. (2004). Glide: A new approach for rapid, accurate docking and scoring. 1. Method and assessment of docking accuracy. J. Med. Chem..

[83] Abrahamsen B., Schneider N., Erichsen M.N., Huynh T.H., Fahlke C., Bunch L., Jensen A.A. (2013). Allosteric modulation of an excitatory amino acid transporter: The subtype-selective inhibitor UCPH-101 exerts sustained inhibition of EAAT1 through an intramonomeric site in the trimerization domain. J. Neurosci..

[84] Chen I., Pant S., Wu Q., Cater R.J., Sobti M., Vandenberg R.J., Stewart A.G., Tajkhorshid E., Font J., Ryan R.M. (2021). Glutamate transporters have a chloride channel with two hydrophobic gates. Nature.

[85] Attwell D., Miller B., Sarantis M. (1993). Arachidonic acid as a messenger in the central nervous system. Semin. Neurosci..

[86] Piomelli D. (1993). Arachidonic acid in cell signaling. Curr. Opin. Cell Biol..

[87] Piomelli D., Greengard P. (1990). Lipoxygenase metabolites of arachidonic acid in neuronal transmembrane signalling. Trends Pharmacol. Sci..

[88] Aid S., Vancassel S., Poumes-Ballihaut C., Chalon S., Guesnet P., Lavialle M. (2003). Effect of a diet-induced n-3 PUFA depletion on cholinergic parameters in the rat hippocampus. J. Lipid Res..

[89] Novak E.M., Dyer R.A., Innis S.M. (2008). High dietary omega-6 fatty acids contribute to reduced docosahexaenoic acid in the developing brain and inhibit secondary neurite growth. Brain Res..

[90] Rapoport S.I. (2013). Translational studies on regulation of brain docosahexaenoic acid (DHA) metabolism in vivo. Prostaglandins Leukot. Essent. Fat. Acids.

[91] Salem N., Litman B., Kim H.Y., Gawrisch K. (2001). Mechanisms of action of docosahexaenoic acid in the nervous system. Lipids.

[92] Moore S.A. (1993). Cerebral endothelium and astrocytes cooperate in supplying docosahexaenoic acid to neurons. Adv. Exp. Med. Biol..

[93] Moore S.A. (2001). Polyunsaturated fatty acid synthesis and release by brain-derived cells in vitro. J. Mol. Neurosci..

[94] Williard D.E., Harmon S.D., Kaduce T.L., Preuss M., Moore S.A., Robbins M.E., Spector A.A. (2001). Docosahexaenoic acid synthesis from n-3 polyunsaturated fatty acids in differentiated rat brain astrocytes. J. Lipid Res..

[95] Bazan N.G. (2003). Synaptic lipid signaling: Significance of polyunsaturated fatty acids and platelet-activating factor. J. Lipid Res..

[96] Green J.T., Orr S.K., Bazinet R.P. (2008). The emerging role of group VI calcium-independent phospholipase A2 in releasing docosahexaenoic acid from brain phospholipids. J. Lipid Res..

[97] Strokin M., Sergeeva M., Reiser G. (2003). Docosahexaenoic acid and arachidonic acid release in rat brain astrocytes is mediated by two separate isoforms of phospholipase A2 and is differently regulated by cyclic AMP and Ca2+. Br. J. Pharmacol..

[98] Strokin M., Sergeeva M., Reiser G. (2007). Prostaglandin synthesis in rat brain astrocytes is under the control of the n-3 docosahexaenoic acid, released by group VIB calcium-independent phospholipase A2. J. Neurochem..

[99] Kuratko C.N., Barrett E.C., Nelson E.B., Salem N. (2013). The relationship of docosahexaenoic acid (DHA) with learning and behavior in healthy children: A review. Nutrients.

[100] Valtcheva S., Venance L. (2019). Control of Long-Term Plasticity by Glutamate Transporters. Front. Synaptic Neurosci..

[101] Tzingounis A.V., Wadiche J.I. (2007). Glutamate transporters: Confining runaway excitation by shaping synaptic transmission. Nat. Rev. Neurosci..

[102] Conti F., Weinberg R.J. (1999). Shaping excitation at glutamatergic synapses. Trends Neurosci..

[103] Rose C.R., Felix L., Zeug A., Dietrich D., Reiner A., Henneberger C. (2017). Astroglial Glutamate Signaling and Uptake in the Hippocampus. Front. Mol. Neurosci..

[104] Fujita S., Ikegaya Y., Nishikawa M., Nishiyama N., Matsuki N. (2001). Docosahexaenoic acid improves long-term potentiation attenuated by phospholipase A(2) inhibitor in rat hippocampal slices. Br. J. Pharmacol..

[105] Mazzocchi-Jones D. (2015). Impaired corticostriatal LTP and depotentiation following iPLA2 inhibition is restored following acute application of DHA. Brain Res. Bull..

